# Exploration on sagittal alignment and clinical outcomes after consecutive three-level hybrid surgery and anterior cervical discectomy and fusion: a minimum of a 5-year follow-up

**DOI:** 10.1186/s13018-020-01589-7

**Published:** 2020-02-26

**Authors:** Shuai Xu, Yan Liang, Guanjie Yu, Zhenqi Zhu, Kaifeng Wang, Haiying Liu

**Affiliations:** Department of Spinal Surgery, Peking University People’s Hospital, Peking University, No. 11 Xizhimen South Street, 100044, Xicheng District, Beijing, People’s Republic of China

**Keywords:** Cervical alignment, Radiological outcomes, Clinical outcomes, Hybrid surgery, Anterior cervical discectomy and fusion, Three-level surgery

## Abstract

**Purpose:**

To compare sagittal alignment and clinical outcomes between three-level hybrid surgery (HS) and anterior cervical discectomy and fusion (ACDF) on cervical spondylotic myelopathy (CSM) over a 5-year follow-up.

**Method:**

The study included 32 patients with ACDF, 36 patients with 1 prosthesis and 2 cages (HS1 group), and 25 cases with 2 prostheses and 1 cage (HS2 group). Alignment parameters included C2–C7 cervical lordosis (CL), C2–C7 sagittal vertical axis (SVA), T1 slope (T1S), and T1S minus CL (T1SCL). Radiographic parameters were range of motion (ROM), upper and lower adjacent ROM (UROM and LROM), and operated-segment lordosis (OPCL), as well as adjacent segment degeneration (ASD). Clinical outcomes included the neck disability index (NDI) and Japanese Orthopedic Association (JOA) score.

**Results:**

Three groups were well-matched in demographics. All groups gained comparable improvement on NDI and JOA (*P* < 0.01). All groups gained CL improvement at the final visit (*P* < 0.05). There were no statistical differences on SVA and T1SCL among the groups and among preoperation, 1 week later, and final follow-up (*P* > 0.05) while T1S improved at 1 week later and final follow-up with HS2. The final change of all alignment parameters among the three groups was of no differences. ROM decreased and OPCL increased in all groups at the final follow-up (*P* < 0.05). UROM and LROM increased with ACDF but kept stable with HS1 and HS2. There was no inter-group difference on the incidence of ASD (*P* > 0.05).

**Conclusion:**

Cervical alignment was comparably improved. HS and ACDF provided identified mid-term efficacy, and it was not necessary to have to use prosthesis on three-level CSM.

## Introduction

Cervical spondylotic myelopathy (CSM) is associated with spinal cord dysfunction that involves the bulging of disks, thickening of soft tissues, and joint laxity [1, 2]. Anterior cervical discectomy and fusion (ACDF), focusing on compression of the ventral aspect of the spinal cord, is a standard and accepted procedure for treating CSM [3, 4]. Although total artificial disk replacement (TDR) has been proven superior to ACDF for motion preservation, controversy still existed as to the ideal surgical approaches that could benefit patients on cervical motion and stability with multilevel CSM [5]. In this regard, hybrid surgery (HS), combining with fusion and arthroplasty technology where appropriate, might be an alternative for treatment with multilevel CSM [6].

Publications have supported identified radiological and clinical outcomes on single- or double-level ACDF and HS [3, 7] while multilevel surgeries, involving more cervical vertebrae, were few studied. Although Kang et al. [6] compared three-level HS and ACDF for cervical disk disease, the conclusion in favor of HS was restricted in radiological parameters and short-term follow. Furthermore, one dynamic-implant combined with two cages might exert different biomechanics from two prostheses combined with one cage [8], which was not emphasized in Kang’s data and a stratified analysis within HS surgeries should be performed.

In addition, it showed ACDF and HS could restore focal lordosis and have an impact on the whole cervical spine alignment, which was the main role in many publications [3, 5] but still debatable in three-level cases. Therefore, the objective of this study was to compare sagittal alignment, radiological, and clinical outcomes between consecutive three-level HS and ACDF on CSM with a minimum of 5-year follow-up.

## Materials and methods

### Participants and procedure selection

A total of 113 patients with CSM enrolled in this retrospective study from February 2007 to September 2013, and all patients have signed informed consent. The inclusion criteria were (1) patients required surgery with uncontrolled symptoms after 6-month conservation treatment, (2) consecutive three-level HS or ACDF was performed, and (3) patients with intact radiographic and clinical outcomes. The exclusion criteria were followed by (1) patients’ radiological parameters were too unclear to measure (*n* = 8), (2) previous cervical spine surgery (*n* = 1), (3) cervical spine fracture or infection (*n* = 1), (4) follow-up < 5 years or incomplete information (*n* = 7), and (5) mortality (*n* = 3).

The target segment performed fusion or arthroplasty was determined by radiographs, CT, or MRI. ACDF could be applied to more severe degenerative segment, and TDR was used to the degenerative segment in accordance with (1) range of motion (ROM) was ≥ 6°, (2) the height loss of intervertebral space was < 80% of the normal adjacent segment, (3) no obvious instability of the segment, (4) no much loss of lordosis, (5) no obvious canal stenosis, and (6) no obvious osteoporosis, but it still lacks consensus of the threshold for (3) to (6) [9].

### Surgical procedure

Each patient was performed ACDF or HS by the same senior surgeon. A right-sided incision and a standard Smith-Robinson approach to the cervical spine were performed. After complete decompression, three PEEK cages were implanted during ACDF procedures without anterior rigid plating (ACDF group) while one artificial disk combined with two stand-alone PEEK cages (HS1 group) or two artificial disks combined with one cage (HS2 group) were implanted in HS. Artificial disks included Prodisc-C (Depuy Synthes, USA) while PEEK cage was MC+ (LDR Medical, France). All patients were instructed to wear soft collar for 2 months after surgery.

### Radiological parameters evaluation

#### Cervical alignment parameters

**Fig. 1:**
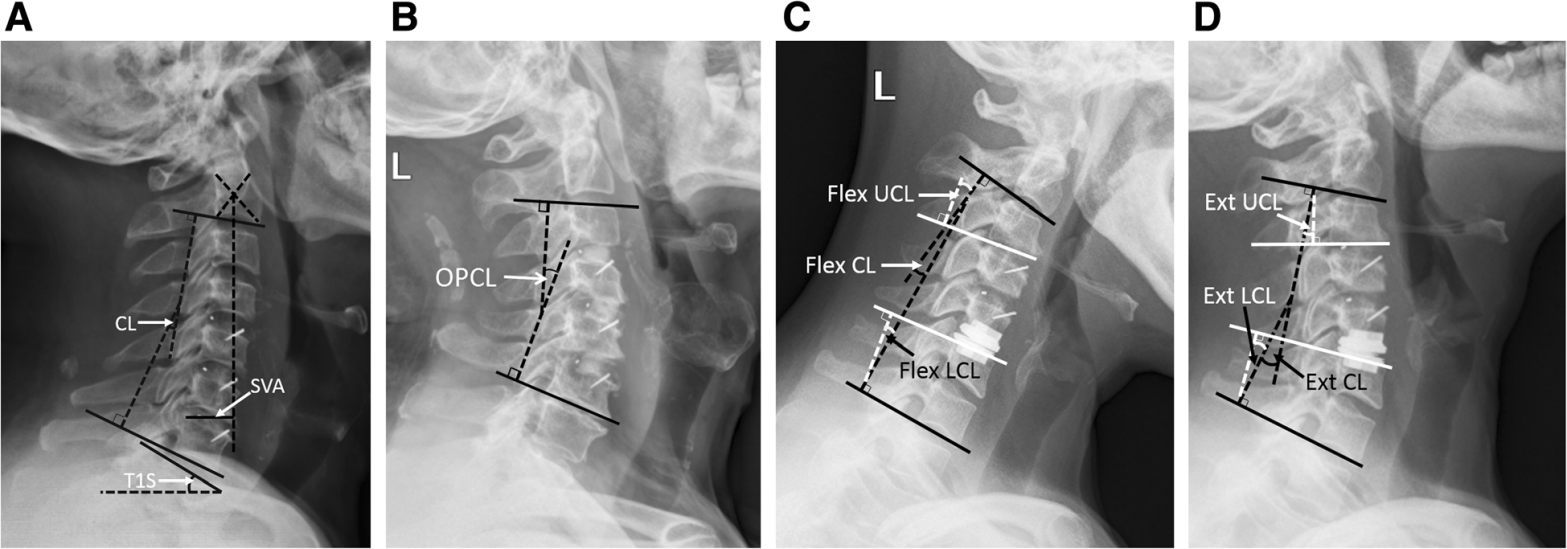
Measurement of cervical alignment and radiological parameters. **a** measurement of CL, SVA, and T1S. **b** Measurement of OPCL. **c** Measurement of radiological parameters on flexion lateral image and **d** on extension lateral image. CL C2–C7 cervical lordosis, SVA C2–C7 sagittal vertical axis, T1S T1 slope, OPCL lordosis of operated segments, UCL lordosis of upper adjacent segment, LCL lordosis of lower adjacent segment

Lateral neutral X-ray was obtained at preoperation, 1 week after surgery, and final follow-up with a minimum of 5 years. Sagittal alignment parameters included C2–C7 cervical lordosis (CL), C2–C7 sagittal vertical axis (SVA), T1 slope (T1S), and T1S minus CL (T1SCL). CL was from lower endplate of C2 to lower endplate of C7; SVA was measured from C2 plumb line to posterior margin of the upper endplate of C7; T1S was from upper endplate of T1 to horizontal line. T1SCL was used to evaluate the cervical sagittal balance (T1SCL ≤ 20°, balance; T1SCL > 20°, imbalance) [10] (Fig. 1).

#### Anatomic measurements and complications evaluation

Lateral flexion-extension X-ray was obtained at preoperation and final follow-up, where ROM, upper and lower adjacent segment ROM (UROM and LROM), and lordosis of operated segments (OPCL) was measured. ROM was defined as the extension angle minus the flexion angle. UROM was the extension angle of upper adjacent functional spinal unit (FSU) minus that of the flexion angle, so was LROM. OPCL was the Cobb angle between the superior endplate of the superior operated vertebrae and the inferior endplate of caudal operated vertebrae (Fig. 1). Radiological adjacent segment degeneration (ASD) was determined by the presence of disk space narrowing > 50%, new or enlarged osteophytes, endplate sclerosis, or increased calcification of the anterior longitudinal ligament [7]. The cage fusion or artificial disk lock was defined as more than 50% of trabecular bridging or no motion (≤ 2°) [11].

### Clinical outcomes assessment

Clinical outcomes included neck disability index (NDI) and Japanese Orthopedic Association (JOA) score, which were both evaluated at preoperation, 1 week after surgery, and final follow-up. The recovery rate (RR) of JOA was calculated by the Hirabayashi method: RR(%) = (PostOP JOA-PreOP JOA)/(17-PreOP JOA) × 100.

### Statistical analysis

Measurement data are expressed as the^−^x ± s. One-way analysis of variance and Kruskal-Wallis test were used to compare variables among ACDF, HS1 and HS2 groups, and among preoperation, 1 week after surgery, and final follow-up. Chi-squared test or Fisher test was performed on dichotomous. The statistical analysis was performed using IBM SPSS Statistics 22.0 (International Business Machines Corporation, Armonk, NY, USA), and statistical significance was defined as *P* < 0.05.

## Results

Eventually, there were 93 participants enrolled with a mean follow-up of 76.4 ± 9.0 m, including 32 patients in the ACDF group, 36 patients in the HS1 group, and 25 cases in the HS2 group. Three groups were well-matched on age, gender, and body mass index (BMI). The operated-segment distribution, operation time, and blood loss were of no difference among groups (*P* > 0.05), but operation time was shorter in ACDF than the HS2 group (*P* = 0.026) (Table 1).

**Table 1:** Demographic characteristics and surgery information among ACDF, HS1, and HS2 groups

	ACDF	HS1	HS2	*P*
Gender (M/F)	17/15	17/19	14/11	0.779
Age (years)	57.2 ± 8.3	55.5 ± 7.5	55.2 ± 12.6	0.695
BMI (kg/m^2^)	24.8 ± 3.4	25.3 ± 3.3	26.0 ± 1.6	0.658
DM (*n*)	3	7	4	0.505
Smoking (*n*)	6	10	5	0.631
Follow-up (months)	74.5 ± 9.6	78.3 ± 8.5	76.0 ± 6.3	0.205
Operated segments				0.295
C3–C6 (*n*)	7	11	4	0.403
C3–C6: CCD/CDC/DCC^a^	/	2/7/2	/	
C3–C6: DDC/DCD/CDD^a^			2/1/1	
C4–C7 (*n*)	25	25	21	
C4–C7: CDC/DCC^a^	/	3/22	/	
C4–C7: DDC/DCD/CDD^a^	/	/	13/7/1	
Operation time (min)	101.1 ± 21.1	107.3 ± 17.8	122.0 ± 13.0	0.063
Blood loss (ml)	71.3 ± 37.1	86.8 ± 73.5	85.0 ± 36.4	0.538

*ACDF* anterior cervical discectomy and fusion, *HS* hybrid surgery, *HS1* one prosthesis and two cages, *HS2* two prostheses and one cage, *M* male, *F* female, *BMI* body mass index, *DM* diabetes mellitus

^a^It means the implantation order from cranial to caudal segments. For example, C3–C6: CCD means artificial disk of C3/4 + artificial disk of C4/5+ peek cage of C5/6

### Radiological parameters among ACDF, HS1, and HS2

#### Cervical alignment parameters

There were no statistical differences on global CL among ACDF, HS1, and HS2 at preoperation, 1 week later, and final follow-up (*P* > 0.05). After surgery, three groups all gained CL improvement, and there were statistical differences at the final visit compared with preoperation (*P* < 0.05). SVA and T1SCL were both lower in the HS1 group than in ACDF before surge while there were no statistical differences among the three groups at pre- and post-operation (*P* > 0.05). The inner-group comparisons also showed no significance on SVA and T1SCL at 1 week and the last visit after surgery (*P* > 0.05). There were T1S improvements at 1 week later and final follow-up in HS2. But T1S were of no statistical differences among the three groups and among preoperation, 1 week later, and final follow-up in the ACDF and HS1 group. In addition, there were no statistical differences on the final change of all cervical alignment parameters among the three groups (*P* > 0.05) (Table 2).

**Table 2:** Comparisons on cervical alignment parameters among ACDF, HS1, and HS2

	ACDF	HS1	HS2	*P*
CL at pre-op (°)	6.8 ± 14.3	12.1 ± 11.0	9.7 ± 15.5	0.231
CL at 1 week later (°)	11.1 ± 10.2	14.7 ± 9.1	16.2 ± 10.0^a^	0.237
CL at final follow-up (°)	12.6 ± 9.0^b^	16.3 ± 7.4^b^	18.9 ± 11.7^b^	0.113
ΔCL (°)	5.9 ± 10.9	4.2 ± 9.3	9.2 ± 12.1	0.538
SVA at pre-op (cm)	2.0 ± 1.0	1.5 ± 1.1	1.8 ± 0.7	0.088
SVA at 1 week later (cm)	2.6 ± 1.3	2.2 ± 1.0	2.4 ± 0.5	0.375
SVA at final follow-up (cm)	1.9 ± 1.2	1.7 ± 1.1	1.9 ± 1.5	0.738
ΔSVA (cm)	− 0.1 ± 1.0	0.3 ± 0.9	0.1 ± 1.4	0.406
T1S at pre-op (°)	24.1 ± 8.4	24.0 ± 8.2	22.1 ± 3.9	0.881
T1S at 1 week later (°)	26.1 ± 8.0	26.7 ± 7.9	28.2 ± 6.8^aa^	0.851
T1S at final follow-up (°)	26.0 ± 7.3	26.7 ± 7.4	26.4 ± 2.6^b^	0.908
ΔT1S (°)	1.9 ± 8.7	2.7 ± 5.9	4.3 ± 2.1	0.745
T1SCL at pre-op (°)	17.3 ± 9.9	11.9 ± 9.7	12.5 ± 13.7	0.081
T1SCL at 1 week later (°)	15.1 ± 8.9	11.9 ± 9.0	12.0 ± 13.3	0.360
T1SCL at final follow-up (°)	13.3 ± 9.9	10.4 ± 7.7	7.2 ± 9.3	0.216
ΔT1SCL (°)	− 4.0 ± 10.0	− 1.5 ± 9.6	− 5.2 ± 13.2	0.506

*ACDF* anterior cervical discectomy and fusion, *HS* hybrid surgery, *HS1* one prosthesis and two cages, *HS2* two prostheses and one cage, *CL* C2–C7 cervical lordosis, *Pre-op* preoperation, *SVA* C2–C7 sagittal vertical axis, *T1S* T1 slope, *T1SCL* T1S minus CL

“Δ” is the change of variable at final follow-up compared to baseline

^a^Significance on parameters between pre-op and 1 week after surgery (*P* < 0.05)

^aa^Significance on parameters between pre-op and 1 week after surgery (*P* < 0.01)

^b^Significance on parameters between pre-op and final follow-up (*P* < 0.05)

#### Anatomic measurements and complications

ROM were of no differences among groups at baseline and final visit, so were their change. While there was a ROM decrease in all groups at final follow-up (*P* < 0.05), UROM and LROM were comparable at baseline among three groups while the two parameters got lower in the HS1 and HS2 groups compared with the ACDF group (*P* < 0.05). UROM and LROM got increased (P < 0.05) in the ACDF group at final follow-up. There was no significance on OPCL among the three groups at preoperation while all groups got comparable improvement at final visit (*P* < 0.05) (Table 3).

**Table 3:** Comparisons on anatomic radiographic parameters and ASD among ACDF, HS1, and HS2

	ACDF	HS1	HS2	*P*
ROM at pre-op (°)	38.6 ± 10.3	44.7 ± 13.4	40.5 ± 10.6	0.153
ROM at final follow-up (°)	24.0 ± 6.3^bb^	29.0 ± 9.1^bb^	32.7 ± 9.9^b^	0.124
ΔROM	− 16.2 ± 10.0	− 15.8 ± 14.3	− 7.8 ± 8.5	0.488
UROM at pre-op (°)	9.8 ± 4.7	9.3 ± 4.4	8.7 ± 3.4	0.867
UROM at final follow-up (°)	11.6 ± 4.0	8.4 ± 3.3	8.5 ± 4.9	0.044
ΔUROM	1.7 ± 7.0	− 1.0 ± 4.7	− 0.3 ± 4.0	0.389
LROM at pre-op (°)	6.9 ± 3.6	6.5 ± 4.3	5.9 ± 3.1	0.858
LROM at final follow-up (°)	9.6 ± 3.3	4.9 ± 3.8	6.4 ± 3.1	0.002
ΔLROM	1.8 ± 6.4^b^	− 2.1 ± 4.0	0.1 ± 2.9	0.078
OPCL at pre-op (°)	3.3 ± 10.9	8.2 ± 8.5	1.7 ± 12.7	0.106
OPCL at final follow-up (°)	9.4 ± 7.0	13.8 ± 7.6	11.9 ± 13.8	0.142
ΔOPCL	6.9 ± 10.0^b^	5.2 ± 8.0^bb^	8.2 ± 7.6^b^	0.688
ASD (*n*)	20	22	17	0.707
Upper ASD (*n*)	6	13	9	0.224
Lower ASD (*n*)	14	14	10	0.916

*ACDF* anterior cervical discectomy and fusion, *HS* hybrid surgery, *HS1* one prosthesis and 2 cages *HS2*, two prostheses and one cage *ROM*, cervical range of motion, *Pre-op*, preoperation, *UROM* ROM of upper adjacent segment, *LROM* ROM of lower adjacent segment *OPCL*, cervical lordosis of operated segment, *ASD* adjacent segment degeneration

“Δ” is the change of variable at final follow-up compared to baseline

^b^Significance on parameters between pre-op and final follow-up (*P* < 0.05)

^bb^Significance on parameters between pre-op and final follow-up (*P* < 0.01)

There was no inter-group difference on the incidence of ASD (*P* > 0.05), so were the respective incidence of upper and lower ASD (Table 3). There was one case that underwent posterior single-door laminoplasty for a severe compression and unsatisfied outcome at 1 month after ACDF while of no secondary surgery in HS1 or HS2. All three groups acquired 100% fused rate in cage-implanted segments at the final visit. However, two segments (2/36) from the HS1 group and one segment (1/50) from the HS2 group planted artificial disks were locked and lost ROM.

### Clinical outcomes among ACDF, HS1, and HS2

There were all no statistical differences on NDI and JOA among the three groups before surgery, at post-operation and final visit (*P* > 0.05). All three groups gained comparable improvement on NDI and JOA after surgery (*P* < 0.01). Besides, NDI and JOA get further improvement at the final visit compared with 1 week after surgery (*P* < 0.05) except JOA of HS2 group (Table 4).

**Table 4:** Comparisons on NDI and JOA among ACDF, HS1, and HS2

	ACDF	HS1	HS2	*P*
NDI at pre-op	38.0 ± 3.0	38.9 ± 3.9	37.9 ± 2.1	0.230
NDI at 1 week later	19.3 ± 6.9	19.9 ± 4.4	19.3 ± 5.1	0.875
NDI at final follow-up	12.5 ± 8.2	10.7 ± 3.3	11.8 ± 2.2	0.163
Δ1 NDI	19.0 ± 6.9	19.9 ± 5.0	18.5 ± 3.7	0.792
Δ2 NDI	25.8 ± 8.2	28.8 ± 5.6	27.2 ± 2.0	0.215
P1 (NDI)	< 0.001	< 0.001	< 0.001	
P2 (NDI)	< 0.001	< 0.001	< 0.001	
P3 (NDI)	< 0.001	< 0.001	0.009	
JOA at pre-op	10.3 ± 1.9	11.0 ± 1.7	11.3 ± 1.5	0.125
JOA at 1 week later	14.6 ± 1.4	14.9 ± 0.8	15.3 ± 0.5	0.433
JOA at final follow-up	15.7 ± 1.9	16.2 ± 1.1	15.8 ± 1.0	0.406
RR1 JOA (%)	63.8 ± 23.5	63.4 ± 13.2	56.3 ± 18.5	0.740
RR2 JOA (%)	81.8 ± 29.4	87.0 ± 18.1	71.0 ± 21.0	0.361
P1 (JOA)	< 0.001	< 0.001	0.002	
P2 (JOA)	< 0.001	< 0.001	0.001	
P3 (JOA)	0.018	< 0.001	0.456	

*NDI* neck disability index, *JOA* Japanese Orthopedic Association score, *ACDF*, anterior cervical discectomy and fusion, *HS* hybrid surgery, *HS1* one prosthesis and 2 cages, *HS2* two prostheses and one cage, *Pre-op* preoperation, *P1* significance between pre-op and 1 week after surgery, *P2* significance between pre-op and final follow-up, *P3* significance between 1 week after surgery and final follow-up

“Δ1” is the change of variable at 1 week after surgery compared to baseline and “Δ2” is the change of variable at final follow-up compared to baseline

## Discussion

The comparison of HS and ACDF has been studied mainly on short-level operation [9, 12]. Xiong et al. [9] compared mid-term outcomes of HS and ACDF with a 6-year visit and concluded HS yielded similar clinical improvement to ACDF and demonstrated better preservation of ROM. Chen et al. [13] performed a short-term study on HS and posterior laminoplasty and showed HS preserved cervical curvature with a lower late complication rate. However, few studies have simultaneously and systematically compared mid-term radiological outcomes and qualification of life between three-level HS and ACDF surgery, let alone subgroup of HS. Cervical alignment has been a hot and debatable issue where studies reported alignment closely related to clinical outcomes [14] while others held ambiguous points [1, 2]. This study firstly demonstrated identified alignment change and clinical efficacy improvement after three-level HS and ACDF surgery on CSM with a mid-term follow-up.

Cervical laminoplasty, sometimes selected for multi-level CSM, can maintain the mobility of the cervical spine while anterior approach could also acquire effective outcomes [13, 15]. However, there have been reported some disadvantages about laminoplasty. The cervical-balance maintaining and reconstruction after both HS and ACDF was, to a great extent, due to the less incision and protection for the posterior cervical muscle-ligament complex. Sakai et al. [16] found postoperative cervical sagittal alignment and balance were maintained after ACDF but deteriorated following laminoplasty by a review on prospective studies. Chen et al. [13] showed HS may preserve cervical curvature with a lower late complication rate than cervical laminoplasty. As an indirect method, the effectiveness of posterior decompression is limited, especially in individuals with absence or reversal of the physiological curvature. In anterior approach surgeries, ACDF and TDR are the most commonly used methods to reconstruct cervical stability in patients with sufficient decompression.

Based on previous research and clinical experience with TDR and ACDF, surgery indications and contraindications have been drafted for treatments [7, 17].TDR was considered a reasonable option with a simple herniated disk without significant joint instability, facet joint degeneration, preoperative ASD, disk calcification, and extensive spinal stenosis. In the case of radiographic signs of instability or no motion at the target levels, with or without facet degeneration, ACDF was achieved [7], which was in consistent with what the criteria we adopted reported by Xiong [9]. Had to admit, selection bias was inevitable in retrospective studies for the different indications for each procedure, and it was unrealistic to undertake randomized controlled trials, even with prospective studies [2]. In that case, although probable with different baseline of parameters, the change of each measurement was introduced in this study to reduce selection bias.

Grasso [7] showed short-term ROM increased in two- to multi-level HS group compared with ACDF-treated patients. A comparison between the two surgeries with double levels showed the mean UROM and LROM were similar preoperatively, but UROM was significantly different at the 3-year follow-up [9]. Lu et al. [18] performed a systematic review that showed that C2–C7 ROM was significantly greater after HS than ACDF, while UROM and LROM were significantly lower. In our study, the decrease of global ROM in the three groups after surgeries might due to more fused intervene on multi-segments with such a long follow-up duration. While it was effective for TDR since the decrease of ROM gradually was relieved from ACDF to HS2 although without statistical significance. A stable UROM and LROM in the HS1 and HS2 groups but larger ones with ACDF indicated an overcompensate ROM on adjacent segment to approximate physical status and an impact on rational-distributed tendency of ROM with HS, which was in line with previous studies.

One of the major concerns regarding ACDF was it could not preserve the normal kinematics of the cervical spine and might result in ASD. Increased motion and intradiscal pressure have been reported in the untreated levels adjacent to fused levels [19]. Accordingly, HS aims to tailor ACDF or TDR to the selected levels for preserving segmental motion of the cervical spine, avoiding long-level fusion, and preventing further ASD [20, 21]. Biomechanical studies have shown increased intra-discal pressure on the adjacent disks after a fusion model [22] and ensuing a higher occurrence of ASD. However, it remained debatable [23] that there was no significance between the two procedures. In our study, radiological ASD with a 64.8% ratio in the three groups was of no inter-group difference during a 5-year follow and no case suffered clinical ASD.

The reason of indifference on ASD might be as follows: Firstly, it was based on small-sample comparison and statistical significance was lightened. Secondly, the impact of artificial disk was weakened in three-level operation particularly in the HS1 group, and the decrease of target-segmental ROM influenced by heterotopic ossification over 5 years impeded the function of artificial disk. Thirdly, ASD might be a natural progress but not totally a iatrogenic outcome, and exceed ROM was not surely inconsistent with ASD [8, 24]. Maldonado et al. [25] published a prospective cohort study comparing ASD after TDR and ACDF. They found that preservation of motion in TDR patients was not associated with a reduction of ASD and concluded that there may be other factors that influence ASD. It has also been reported that multilevel ACDF do not significantly increase the risk of ASD at the C7-T1 level contrasted to HS, and ASD occurred mainly in the middle region of cervical spine [26], which was in line with this study of no difference on ASD since the middle region had escaped being adjacent segment with three-level surgery.

OPCL recovery was beneficial to cervical curve maintaining because three-level region occupied most proportion of the overall cervical spine, and OPCL correction was more suitable for stress distribution physiologically [10]. In addition, cervical alignment could be reconstructed through release of anterior tissue, removal of osteophyte, repairment of endplate bed, the pattern and the bonding of implants, reported by Di Martino et al. [11]. Meanwhile, they found a straight cervical spine was related to increased SVA and larger T1SCL, which was shown in our data before surgery. ACDF was more likely to be selected for patients with straight cervical spine for its indications with more facet degeneration. Thus, the baseline of SVA and T1SCL was larger in the ACDF group than in HS. However, most cases (90.1%) gained cervical balance after surgery, and it showed comparable capacity between HS and ACDF on cervical alignment reconstruction.

Neurologic function and quality of life reflected by NDI and JOA of all patents were improved after surgeries and a further promotion at final follow-up, which was attributed to a better adaption of postoperative status, the further edema-elimination and a progressed repairment of nerve root as well as the regular functional training [7, 27]. The key objective of either HS or ACDF surgery was to remove compression of spinal cord and neurological function recovery. So in our series, the three groups showed a mid-term and safe efficacy in the treatment of CSM. Therefore, regardless of the surgical-indications of ACDF and TDR, taking cost and medical insurance in consideration, we suggested ACDF was enough for three-level CSM, and it was not necessary to use prosthesis.

There were still some limitations in this study. Firstly, the sample of both groups was little. Probably, a larger population could support a strong verification with a cohort study. Then, there was no subgroup analysis on operated-segment region (C3–C6/C4–C7), and the types of artificial disks. Finally, only the patients with CSM were included, and the conclusion might not be suitable for other cervical spine disease such as spondylotic radiculopathy.

## Conclusions

Cervical alignment was comparably improved, and most patients gained cervical balance by HS and ACDF through an over 5-year follow-up. Global cervical ROM all decreased with insignificant change in the three groups. ROM of adjacent segments increased in ACDF group while kept stable in the HS1 and HS2 groups, but the incidence of ASD was of no difference. In total, HS and ACDF provided an identified and mid-term efficacy. Therefore, it was not necessary to use prosthesis in the treatment of three-level CSM.

## Data Availability

The datasets used and/or analyzed during the current study are available from the corresponding author on reasonable request.

## Abbreviations

ACDF: Anterior cervical discectomy and fusion
ASD: Adjacent segment degeneration
BMI: Body mass index
CL: C2–C7 cervical lordosis
CSM: Cervical spondylotic myelopathy
FSU: Functional spinal unit
HS: Hybrid surgery
JOA: Japanese Orthopedic Association
NDI: Neck disability index
OPCL: Lordosis of operated segments
ROM: Range of motion
RR: Recovery rate
SVA: Sagittal vertical axis
T1S: T1 slope
T1SCL: T1S minus CL
TDR: Total artificial disk replacement
